# The relationship between the timing of lung surgery and postoperative pulmonary complications in patients after SARS-CoV-2 infection: a prospective cohort study

**DOI:** 10.3389/fmed.2025.1640475

**Published:** 2025-07-28

**Authors:** Dawei Yang, Min Li, Xianning Duan, Fuhai Ji, Jianyou Zhang

**Affiliations:** ^1^Department of Anesthesiology, Affiliated Hospital of Yangzhou University, Yangzhou University, Yangzhou, China; ^2^Department of Anesthesiology, The First Affiliated Hospital of Soochow University, Suzhou, China

**Keywords:** SARS-CoV-2, lung surgery, postoperative pulmonary complications, COVID-19, prospective cohort study

## Abstract

**Background:**

Patients with a positive test for SARS-CoV-2 prior to elective surgery early in the pandemic have an elevated risk of perioperative mortality and pulmonary complications. Post-SARS-CoV-2 infection, pulmonary sequelae persist beyond the acute stage, necessitating recovery periods spanning months or even longer. Our study aimed to explore the correlation between the timing of thoracoscopic lung surgery and postoperative pulmonary complications (PPCs) in patients with a history of SARS-CoV-2 infection.

**Methods:**

We conducted a prospective cohort study, enrolling patients scheduled for elective thoracoscopic partial lung resection. Participants were categorized into two groups based on the duration since their SARS-CoV-2 infection: 5–10 weeks and 11–16 weeks. A total of 68 patients were included, with 34 in each group. The information about SARS-CoV-2 infection were collected; IL-6 and TNF-*α* levels at 2 h, 1 d, and 2 d after surgery and the WBC count and CRP level in blood at 1 d and 2 d after surgery were analyzed; and PPCs and length of hospitalization were recorded. A logistic regression model was employed to assess the relationship between the timing of lung surgery and PPCs in patients post SARS-CoV-2 infection.

**Results:**

Compared with the 5-10-week group, in the 11-16-week group, the levels of IL-6 and TNF-*α* at 2 h, 1 d, and 2 d after surgery were significantly lower, the WBC count and CRP levels in blood at 1 d and 2 d after surgery were significantly lower, the numbers of PPCs and lung infections were significantly lower, and the length of hospitalization was significantly shorter. Multivariate logistic regression analysis revealed that the time interval from surgery to SARS-CoV-2 infection, persistent preoperative symptoms, preoperative difficulty breathing and WBC count at 1 d after surgery were independent risk factors for PPCs.

**Conclusion:**

Patients infected with SARS-CoV-2 who underwent thoracoscopic lung surgery within 5 to 10 weeks after infection had a higher risk of PPCs than those who had surgery at 11 to 16 weeks post-infection.

**Clinical trial registration:**

https://clinicaltrials.gov/, ChiCTR2300071539.

## 1 Introduction

Since the coronavirus disease 2019 (COVID-19) pandemic began in late 2019, SARS-CoV-2 has infected hundreds of millions of people worldwide. After 3 years of stringent prevention and control measures, China adjusted its strategy and ended the dynamic COVID-zero policy in December 2022 (1–3). With the easing of COVID-19 restrictions, the prevalence of SARS-CoV-2 infection in the population may increase; therefore, it is necessary to explore the impact of SARS-CoV-2 infection during the perioperative period in china.

The inflammatory storm triggered by SARS-CoV-2 invades many organs, including the lungs (4), brain (5), and heart (6). Even after the virus has been cleared, individuals who have been infected may still have a variety of residual symptoms that can persist for weeks or months (7). Following the viral challenge and inflammatory storm during the acute phase of infection, most individuals who survive an infection with SARS-CoV-2 suffer from persistent lung injuries. These injuries encompass chronic lung inflammation, pulmonary fibrosis, diffusion dysfunction, and pulmonary embolism, which all require months or even longer for recovery (8). Pulmonary complications are prone to occur after lung surgery, with an incidence rate of approximately 33% for thoracoscopic surgery (9), and postoperative pulmonary complications (PPCs) are closely related to the recovery process for and economic burden on patients (10). Patients with a positive test for SARS-CoV-2 prior to elective surgery early in the pandemic have an elevated risk of perioperative mortality and pulmonary complications (11). The effect of the timing of lung surgery after SARS-CoV-2 infection on postoperative outcomes remains unclear. The purpose of this prospective study on patient’s post-SARS-CoV-2 infection was to investigate the effect of the timing of thoracoscopic lung surgery after SARS-CoV-2 infection on PPCs, aiming to offer guidance for clinical practice.

## 2 Materials and methods

### 2.1 Study design

This study was a prospective cohort study reported according to the Consolidated Standards of Reporting Trials (CONSORT). This study was approved by the Ethics Committee of the Affiliated Hospital of Yangzhou University [approval number: 2022-YKL12- (Lesson 01)]. All study participants provided written informed consent without any deviation from the principles of the Declaration of Helsinki.

### 2.2 Study population and baseline characteristics

Patients who underwent elective thoracoscopic partial lung resection in our hospital between January 2023 and May 2023 were eligible for inclusion. All patients had a history of reverse transcription-polymerase chain reaction (RT-PCR)-positive throat swabs. The inclusion criteria were as follows: patients with a single SARS-CoV-2 infection after December 2022; American Society of Anaesthesiologists (ASA) grade I-II; age of 25–75 years; body mass index (BMI) of 18.0–30.0 kg/m^2^; and no vital organ dysfunction. The exclusion criteria were as follows: preoperative lung infection, ≥ moderate anemia, hypoproteinaemia, hypoxemia, water, electrolyte, and acid–base balance disorders, coagulation dysfunction, history of radiotherapy, chemotherapy and immunotherapy, chronic lung diseases, history of asthma, and history of thoracic surgery.

### 2.3 Allocation

**Figure 1:**
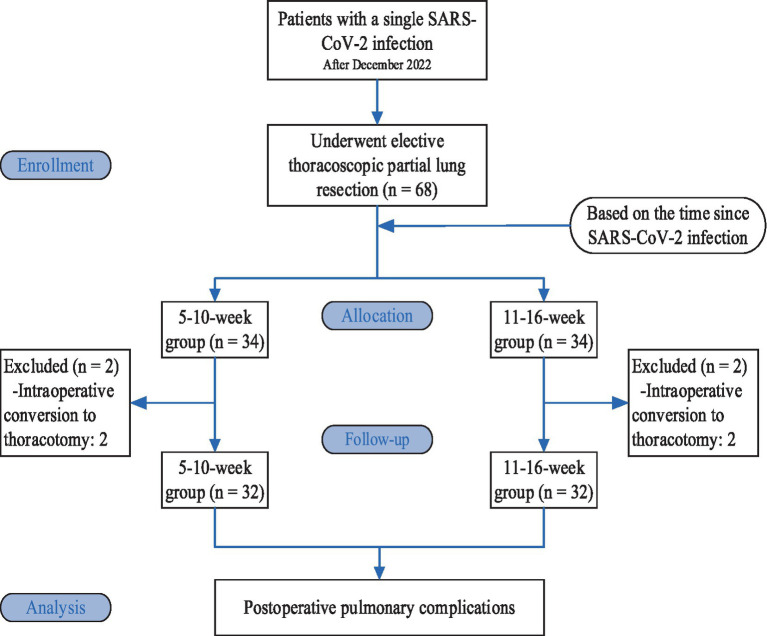
Consolidated standards of reporting trials (CONSORT) flow diagram.

Based on the time since SARS-CoV-2 infection, the patients were divided into a 5-10-week group and an 11-16-week group, with 34 patients in each group. Two patients in each group were excluded from the study due to intraoperative conversion to thoracotomy; therefore, there were 32 patients in each group (Figure 1).

### 2.4 Study procedures

All patients refrained from drinking and eating before surgery. Ultrasound-guided catheterization of the radial artery and the right internal jugular vein was performed. Mean arterial pressure, heart rate, nasopharyngeal temperature, oxygen saturation, and central venous pressure were monitored, and the Narcotrend index (NI) values were monitored using a Narcotrend monitor (MT MonitorTechnik GmbH, Germany). Anesthesia was induced by intravenous bolus injection of 0.05 mg/kg midazolam, 1.5–2.0 mg/kg propofol, 0.5 μg/kg sufentanil, and 0.15 mg/kg cisatracurium. After achieving satisfactory muscle relaxation, a double-lumen endobronchial tube was inserted, and a fibreoptic bronchoscopy was used for positioning. Volume-controlled ventilation was performed with 5 cmH_2_O (1 cmH_2_O = 0.098 kPa) PEEP. The tidal volume (calculated using adjusted body weight) was as follows: 8 mL/kg during two-lung ventilation (TLV) and 6 mL/kg during one-lung ventilation (OLV). The FiO_2_ was set at 100%, the I: E ratio was 1:2, and the nonventilated side of the dual-lumen tube was open to the atmosphere during OLV. The respiratory rate was adjusted to maintain P_ET_CO_2_ at 35–45 mmHg (1 mmHg = 0.133 kPa). Anesthesia was maintained by intravenous pumping of propofol 5 mg·kg^−1^·h^−1^, remifentanil 0.2 μg·kg^−1^·min^−1^, and cisatracurium 0.1 mg·kg^−1^·h^−1^. The doses of propofol and remifentanil were adjusted to maintain the NI at 26–46 and the MAP within ±20% of the basal blood pressure. Intraoperatively, the compound electrolyte solution was infused at 10 mL·kg^−1^·h^−1^, and the volume of fluid replacement was adjusted to maintain the CVP. If the amount of intraoperative blood loss exceeded 500 mL, an equal amount of colloid and vasoactive drugs was administered to maintain haemodynamic stability, and blood product transfusion was considered based on the patient’s hemoglobin concentration and underlying diseases. A warming blanket was used to prevent hypothermia.

### 2.5 Data collection

The primary outcome indicator was the incidence of PPCs (lung infection, respiratory failure, pleural effusion, atelectasis, pneumothorax, and bronchospasm) during hospitalization. For secondary outcome indicators, venous blood samples (3 mL for each) were collected before surgery and at 2 h, 1 d, and 2 d after surgery. These were then centrifuged for 10 min (at 3000 r/min with a centrifugal radius of 10 cm), and the supernatant was collected and stored in a freezer at −80°C. Serum interleukin (IL)-6 and tumor necrosis factor (TNF)-*α* levels were assessed by enzyme-linked immunosorbent assay (ELISA). The white blood cell (WBC) count and C-reactive protein (CRP) level were assessed preoperatively and 1 d and 2 d after surgery. Additional data included general information, hospitalization during the acute stage of SARS-CoV-2 infection, persistent symptoms from the SARS-CoV-2 infection prior to surgery, dyspnea before surgery, and the length of hospitalization stay.

PPCs were defined as follows. According to the European Perioperative Clinical Outcome (EPCO) definitions in 2015 (12), a diagnosis of PPC can be made if any of the following is met: ① lung infection - a suspected respiratory infection requiring antibiotic treatment and meeting at least one of the following criteria: new or progressive expectoration; new or progressive pulmonary infiltration shadows; fever; or WBC > 12 × 10^9^/L; ② respiratory failure - partial pressure of oxygen in the arterial blood (PaO_2_) < 60 mmHg, PaO_2_/FiO_2_ < 300 mmHg, or SpO_2_ < 90% during inspiration, thus requiring oxygen therapy; ③ thoracic cavity effusion confirmed by imaging; ④ atelectasis confirmed by imaging; ⑤ pneumothorax confirmed by imaging; and ⑥ bronchospasm - new wheezing sound necessitating the use of a bronchodilator.

### 2.6 Statistical analysis

This was a prospective cohort study. The sample size was calculated based on the incidence of PPCs in the 5-10-week and 11-16-week groups (68.8% vs. 31.3%) from a pilot experiment. With *α* = 0.05, *β* = 0.2, and a loss to follow-up rate of 20%, at least 30 patients were needed in each group. 34 patients were enrolled in each group in this study. SPSS 23.0 software was used for statistical analysis. Normally distributed measurement data are expressed as the mean ± standard deviation, and intergroup comparisons of measurement data were performed using an independent samples *t* test. Measurement data with a non-normal distribution were expressed as the median (quartile) [*M* (*Q1*, *Q3*)], and intergroup comparisons were performed using the Mann–Whitney U test. Count data were compared using the chi-squaretest, and if the theoretical frequency was less than 5, Fisher’s exact probability test was used. Variables with *p* < 0.05 and clinically significant variables in the univariate analysis were included in the logistic regression analysis. *p* < 0.05 was considered statistically significant.

## 3 Results

### 3.1 Comparison of general conditions

There were no significant differences between the two groups of patients in sex, age, BMI, ASA grade, pulmonary function, treatment of patients in the acute stage of SARS-CoV-2 infection, type of surgery, lung collapse, OLV duration, surgery duration, amount of blood loss, and volume of fluid replacement. Persistent symptoms of SARS-CoV-2 infection and preoperative difficulty breathing were significantly lower in the 11-16-week group (*p* < 0.05; Table 1).

**Table 1:** Comparison of general conditions (*n* = 32).

Indicators	5-10-week group	11-16-week group	*χ*^2^/*t* value	*p* value
Male/female	12/20	15/17	0.577	0.448
Age (years)	58.78 ± 11.00	61.03 ± 8.25	0.925	0.358
BMI (kg/m^2^)	23.98 ± 2.62	24.37 ± 2.68	0.594	0.554
ASA class I/II	10/22	9/23	0.075	0.784
Smoking history [patients (%)]	6 (18.8)	5 (15.6)	0.11	0.74
FEV1 (% estimated value)	95.93 ± 14.14	90.91 ± 13.73	1.441	0.155
FVC (% estimated value)	96.06 ± 14.80	94.17 ± 11.44	0.571	0.57
FEV1/FVC (%)	81.49 ± 6.88	78.65 ± 7.40	1.588	0.117
Hospitalization during the acute stage Yes/No	1/31	2/30		1
With/without persistent symptoms	14/18	6/26*	4.655	0.031
Preoperative difficulty breathing (Yes/No)	8/24	2/30*		0.041
Type of surgery [patients (%)]			0.654	0.721
Lobe of lung	20 (62.5)	23 (71.9)		
Lung segment	5 (15.6)	4 (12.5)		
Wedge resection	7 (21.9)	5 (15.6)		
Collapsed lung left/right	14/18	13/19	0.064	0.8
OLV time (min)	84.64 ± 34.17	83.22 ± 34.33	0.166	0.869
Operative time (min)	107.34 ± 33.67	100.31 ± 34.66	0.823	0.414
Volume of blood loss (ml)	82.50 ± 34.92	77.81 ± 29.04	0.584	0.561
Volume of fluid replacement (ml)	1184.38 ± 243.77	1218.75 ± 197.46	0.62	0.538

Data are expressed as means ± SD. BMI, body mass index; ASA, American Society of Anesthesiologists; FEV1, Forced Expiratory Volume in 1 s; FVC, Forced Vital Capacity; OLV, one-lung ventilation; Compared with the 5-10-week group, *p < 0.05.

### 3.2 Comparison of inflammatory indicators

The levels of IL-6 and TNF-*α* at 2 h, 1 d, and 2 d after surgery were significantly lower in the 11-16-week group than in the 5-10-week group (*p* < 0.05), as were the WBC count and CRP level at 1 d and 2 d after surgery (*p* < 0.05; Table 2).

**Table 2:** Comparison of inflammatory indicators (*n* = 32).

Indicators	5-10-week group	11-16-week group	Z/*t* value	*p* value
IL-6 [pg/mL, *M* (*Q*_1_, *Q*_3_)]				
Preoperative	2.88 (2.21,4.49)	2.48 (2.03,3.56)	−1.259	0.208
2 h after surgery	57.46 (30.43,79.30)	34.10 (19.83,61.13)^a^	−2.216	0.027
1 d after surgery	27.75 (15.98,64.88)	20.65 (7.88,27.95)^a^	−2.377	0.017
2 d after surgery	31.15 (19.57,48.68)	16.05 (8.51,30.51)^a^	−2.726	0.006
TNF-α [pg/mL, M (Q1, Q3)]				
Preoperative	6.55 (5.16,8.86)	6.29 (5.11,7.36)	−0.839	0.401
2 h after surgery	8.65 (6.00,9.96)	6.32 (5.62,7.21)^a^	−2.612	0.009
1 d after surgery	8.42 (5.59,9.76)	6.14 (5.33,7.17)^a^	−2.383	0.017
2 d after surgery	7.44 (6.38,9.06)	6.36 (5.20,7.59)^a^	−2.397	0.017
WBC count (×10^9^/L, means ± SD)				
Preoperative	5.85 ± 2.01	5.52 ± 1.56	0.745	0.459
1 d after surgery	10.72 ± 2.51	9.46 ± 2.31^a^	2.088	0.041
2 d after surgery	8.94 ± 2.28	7.45 ± 1.62^a^	3.013	0.004
CRP (mg/L, means ± SD)				
Preoperative	2.72 ± 2.14	2.27 ± 2.65	0.734	0.465
1 d after surgery	33.76 ± 13.81	22.01 ± 12.21^a^	3.605	0.001
2 d after surgery	46.24 ± 28.88	30.37 ± 27.01^a^	2.27	0.027

Data with a non-normal distribution were expressed as the median (quartile) [M (Q1, Q3)], Normally distributed measurement data are expressed as the means ± SD. WBC, White Blood Cell; CRP, C-Reactive Protein; Compared with the 5-10-week group, ^a^*p* < 0.05.

### 3.3 Comparison of PPCs and length of hospitalization

The number lung infections was significantly lower in the 11-16-week group than in the 5-10-week group (*p* < 0.05), and the length of hospitalization was significantly shorter (*p* < 0.05; Table 3).

**Table 3:** Comparison of PPCs and length of hospitalization (*n* = 32).

Indicators	5-10-week group	11-16-week group	χ^2^/*t* value	*P* value
PPCs [patients (%)]	22 (68.8)	13 (40.6)^b^	5.107	0.024
Occurrence of PPCs [patients (%)]				
Lung infection	19 (59.4)	11 (34.4)^b^	4.016	0.045
Respiratory failure	0	0		
Pleural effusion	5 (15.6)	3 (9.4)		0.708
Atelectasis	2 (6.3)	0		0.492
Pneumothorax	1 (3.1)	1 (3.1)		1
Bronchospasm	0	0		
Length of hospitalization (d, means ± SD)	7.22 ± 2.27	6.22 ± 1.52^b^	2.073	0.042

Data expressed as number of patients (%). PPCs, postoperative pulmonary complications; Compared with the 5-10-week group, ^b^*p* < 0.05.

### 3.4 Logistic regression analysis of PPCs

Variables with *p* < 0.05 and clinically significant variables in the univariate analysis were included in the logistic regression analysis. The variables included in the multivariate regression analysis were categorical variables (time interval from surgery to SARS-CoV-2 infection, presence of persistent preoperative symptoms, and presence of preoperative dyspnea) and a continuous variable (WBC count at 1 d after surgery). The results showed that time interval from surgery to SARS-CoV-2 infection (OR = 1.754, 95% CI: 1.509–2.038, *p* < 0.001), presence of persistent symptoms before surgery (OR = 2.523, 95% CI: 2.047–3.110, *p* < 0.001), preoperative dyspnea (OR = 1.875, 95% CI: 1.406–2.500, *p* < 0.001), and WBC count at 1 d after surgery (OR = 0.676, 95% CI: 0.651–0.701, *p* < 0.001) were independent risk factors for PPCs (Table 4).

**Table 4:** Logistic regression analysis of PPCs.

Indicators	Partial regression coefficient	Standard error	Wald	*p* value	OR (95% CI)
Univariate analysis					
Time interval from surgery to SARS-CoV-2 infection	1.121	0.068	272.123	<0.001	3.068 (2.686–3.505)
Persistent COVID-19 symptoms	1.002	0.075	179.239	<0.001	2.724 (2.353–3.155)
Dyspnea	1.493	0.113	174.345	<0.001	4.449 (3.565–5.552)
WBC count at 1 d after surgery	−0.378	0.018	459.474	<0.001	0.685 (0.662,0.709)
Multivariate analysis					
Time interval from surgery to SARS-CoV-2 infection	0.562	0.077	53.786	<0.001	1.754 (1.509–2.038)
Persistent COVID-19 symptoms	0.925	0.107	75.132	<0.001	2.523 (2.047–3.110)
Dyspnea	0.629	0.147	18.362	<0.001	1.875 (1.406–2.500)
WBC count at 1 d after surgery	−0.392	0.019	419.536	<0.001	0.676 (0.651–0.701)

## 4 Discussion

In this study, quality was strictly controlled, the experimental methods were standardized, and all included patients had a single SARS-CoV-2 infection to avoid confounding effects caused by multiple SARS-CoV-2 infections. EPCO criteria were the most widely used assessment tool for PPCs (13). Firstly, due to the large number of medical staff being infected for the first time during the COVID-19 pandemic, the symptoms were obvious and they required rest. Additionally, it was not recommended to perform elective surgeries within 1 month after the patient’s infection. Consequently, our center began to perform elective surgeries 1 month after the COVID-19 pandemic in the region. Real-world research indicates that during the period when the dominant variant Omicron of the SARS-CoV-2 was prevalent (the main strain during our study period), the risk of re-infection within the first 3 months was 3.31%, and it gradually increased thereafter (14). The efficacy of mixed immune protection significantly decreased after 4–6 months (15, 16). Therefore, we set 16 weeks after infection as the termination point for the study to avoid including patients with secondary infections and to ensure the consistency of the study. Moreover, considering the previous surgical volume of our center, we divided the study into two intervals, specifically 5–10 weeks and 11–16 weeks. Thus, the sample size could be sufficient to meet the statistical requirements.

The results of this study showed that the incidence of PPCs, the length of hospitalization, and levels of inflammation were significantly reduced in patients who underwent thoracoscopic lung surgery 11–16 weeks following SARS-CoV-2 infection compared to those who had surgery 5–10 weeks post-infection. An international multicentre study revealed that lung complications and mortality within 30 days after surgery increased when the procedure was conducted within 6 weeks of SARS-CoV-2 infection, with no significant effect when surgery was performed beyond 7 weeks afterinfection (17). A single-center retrospective study involving 7,927 patients confirmed that the risk of postoperative complications roughly decreases as the time interval between surgery and SARS-CoV-2 infection lengthens (18). A prospective multicenter cohort study in China, involving 2,081 patients found that thoracic surgery for lung cancer performed 4–7 weeks after SARS-CoV-2 infection was associated with an increased risk of 30-day morbidity, whereas surgery performed ≥8 weeks post-infection did not increase this risk (19). Although the time nodes adopted for group division in the aforementioned studies slightly differ from the settings of this research, the results consistently indicate that delayed surgery after SARS-CoV-2 infection can lead to better postoperative recovery outcomes for patients.

Patients infected with SARS-CoV-2 may develop pulmonary sequelae, including persistent diffusion impairment and imaging changes, such as ground-glass opacity (GGO) and pulmonary fibrosis (20). The follow-up of hospitalized patients 4 months after infection showed that 16% experienced difficulty breathing, and lung computed tomography (CT) scans of those with moderate to severe infection showed GGOs in 63% of patients and pulmonary fibrosis in 19.3% (21). An inflammatory storm during the acute infection stage leads to extensive diffuse alveolar damage and extensive lung destruction, which triggers fibrous proliferation, and progression into long COVID-19 results in continuous low-grade systemic inflammation and inflammatory cell infiltration in the lungs, leading to chronic lung inflammation (22) and potentially inducing bacterial colonization and secondary infection (23). In this study, there were no significant differences in the preoperative inflammatory indicators between the 11-16-week and 5-10-week groups, a finding that may be related to the short interval. Follow-up chest CT of 205 patients after SARS-CoV-2 infection suggested that most patients, including many with mild cases, developed GGOs at 1 month after infection and that GGOs gradually resolved 6 months after infection; however, pulmonary fibrosis did not improve, and some patients continued to present with small airway injury (24). Additionally, analyses of lung function evolution in COVID-19 patients showed that lung function gradually improved over time (at 3, 6, and 12 months) (25). The gradual resolution of GGOs and improvements in lung function 1 month after SARS-CoV-2 infection indicate that delaying surgery can provide time for lung condition improvements, which is consistent with the results of this study.

In this study, the number of patients experiencing persistent symptoms was significantly lower in the 11-16-week group compared to the 5-10-week group, aligning with the findings by Whitaker et al. (26). Persistent symptoms following SARS-CoV-2 infection may increase the incidence of PPCs (17). Moreover, the number of patients reporting difficulty breathing was significantly lower in the 11-16-week group than in the 5-10-week group, indicating that breathing difficulties diminish over time, a trend also observed by Wu et al. (25). Difficulty breathing is one of the major sequelae of SARS-CoV-2 infection, occurring in 8–14% of non-hospitalized patients (27), is related to small airway obstruction, pulmonary fibrosis and muscle dysfunction (28), affects postoperative sputum evacuation, and increases the risk of lung infection.

This study has several limitations. First, the sample size was relatively small and the study was conducted at a single center, which suggests that the findings should be validated through larger, prospective multicenter studies. Second, most of the included patients had mild disease manifestations; therefore, the generalizability of the conclusions to more severe patient populations may be limited. Third, preoperative baseline data did not include assessments of respiratory function impairment caused by COVID-19 infection or CT imaging features such as ground-glass opacities and fibrosis. These factors, which are closely associated with post-COVID sequelae, could potentially influence postoperative pulmonary outcomes. However, this study systematically collected and quantitatively analyzed preoperative pulmonary function test results and post-COVID sequelae (e.g., dyspnea symptoms) to ensure consistency across groups, and comprehensively evaluated the association between these functional changes and postoperative pulmonary complications. Fourth, the study focused only on short-term perioperative outcomes; hence, the long-term prognostic implications require further investigation.

Although the panic caused by the COVID-19 pandemic has gradually subsided, the virus remains present and new cases continue to emerge. This study aims to provide guidance for determining the appropriate timing of surgical interventions in clinical practice and offers valuable insights that may be applicable in the event of similar epidemics in the future.

## 5 Conclusion

In summary, patients infected with SARS-CoV-2 who underwent thoracoscopic lung surgery within 5 to 10 weeks after infection had a higher risk of PPCs than those who underwent surgery at 11 to 16 weeks. Postponing surgery can lead to better physical conditions and reduce the occurrence of PPCs and the length of hospitalization stay. However, tumor surgeries are typically semi-elective surgeries, and the optimal timing for surgery should be determined based on a comprehensive evaluation of each patient’s conditions.

## Data Availability

The raw data supporting the conclusions of this article will be made available by the authors, without undue reservation.
